# Enhanced Stability of Dopamine Delivery via Hydrogel with Integrated Graphene

**DOI:** 10.3390/jfb14120558

**Published:** 2023-11-23

**Authors:** Cristina Mormile, Ocsana Opriș, Stefano Bellucci, Ildiko Lung, Irina Kacso, Alexandru Turza, Matteo La Pietra, Cristian Vacacela Gomez, Adina Stegarescu, Maria-Loredana Soran

**Affiliations:** 1 National Institute for Research and Development of Isotopic and Molecular Technologies, 67-103 Donat, 400293 Cluj-Napoca, Romania; mormile.1971077@studenti.uniroma1.it (C.M.); ocsana.opris@itim-cj.ro (O.O.); irina.kacso@itim-cj.ro (I.K.); alexandru.turza@itim-cj.ro (A.T.); adina.stegarescu@itim-cj.ro (A.S.); loredana.soran@itim-cj.ro (M.-L.S.); 2 Faculty of Chemistry, Sapienza University of Rome, P. le Aldo Moro 5, 00185 Rome, Italy; 3 INFN—National Laboratories of Frascati, Via Enrico Fermi 54, 00044 Frascati, Italy; matteo.lapietra@lnf.infn.it (M.L.P.); cristianisaac.vacacelagomez@fis.unical.it (C.V.G.); 4 Department of Information Engineering, Polytechnic University of Marche, Via Brecce Bianche 12, 60131 Ancona, Italy

**Keywords:** hydrogels, graphene, drug delivery, dopamine

## Abstract

The synthesis of graphene-based materials for drug delivery represents an area of active research, and the use of graphene in drug delivery systems is promising due to its unique properties. Thus, in the present work, we discuss the potential of few-layer graphene in a hydrogel system for dopamine release. The hydrogels are frequently used for these systems for their special physico-chemical properties, which can ensure that the drug is effectively released in time. However, the release from such structures is mostly determined by diffusion alone, and to overcome this restriction, the hydrogel can be “improved” with nanoscale fillers like graphene. The release kinetics of the composite obtained were analyzed to better understand how the use of graphene, instead of the more common graphene oxide (GO) and reduced graphene oxide (rGO), affects the characteristics of the system. Thus, the systems developed in this study consist of three main components: biopolymer, graphene, and dopamine. The hydrogels with graphene were prepared by combining two different solutions, one with polyacrylic acid and agarose and one with graphene prepared by the exfoliation method with microwave irradiation. The drug delivery systems were developed by adding dopamine to the obtained hydrogels. After 24 h of release, the presence of dopamine was observed, demonstrating that the system developed can slow down the drug’s degradation because of the interactions with the graphene nanoplates and the polymer matrix.

## 1. Introduction

Smart drug delivery systems (DDSs) represent a milestone in the development of effective therapeutic approaches in modern healthcare. Undoubtedly, researchers have focused on the tunable pharmacokinetics and the effectiveness of released active principles, generating a wide range of polymer-based biomaterials. Among them, hydrogels are generally recognized as tailored three-dimensional (3D) networks to achieve sustained drug release [1].

Hydrogels are water-swollen (bio)polymeric materials that maintain a distinct 3D structure due to the crosslinking of the polymeric chains [2]. Nowadays, the preoccupation with using natural materials for drug development has increased. They have been used for biomedical purposes due to their excellent mechanical properties, good biocompatibility, and biodegradability, in addition to having a physiochemistry which is comparable to the native extracellular matrix, which can act as a supporting material for a drug delivery system [3,4,5]. Due to these properties, hydrogels can be considered smart materials, meaning that they are capable of responding to multiple triggers, and this gives the hydrogel tailoring properties for the desired application [6]. Besides drug delivery, the applications of hydrogels in the biomedical field are for tissue engineering as scaffolds. They are attractive scaffolding materials thanks to the highly swollen network structure and the capacity to encapsulate cells and bioactive molecules. A variety of polymers, which include natural, synthetic, and hybrid polymers, have been used to produce hydrogels by chemical or physical crosslinking or by both [7]. Chitosan [8,9,10], starch [11,12], carboxymethyl cellulose [13], carrageenan [14], and tragacanth gum [15] are biopolymers and their derivatives have been used for applications in sustained- and controlled-release drug delivery systems. These polymers present less toxicity, biocompatibility, and biodegradability, by which they are degraded via the action of enzymes [16,17]. Also, alginate, which is an anionic polysaccharide, demonstrated a growing appreciation for active delivery with its extraordinary physical and biological characteristics [18]. Alginates are naturally occurring polysaccharides obtained mainly from marine brown algae and were preferentially used as gel beads in the delivery of low-solubility or macromolecular drugs [19,20]. Among different alginates, sodium alginate is one of the most studied in the pharmaceutical domain for drug delivery applications [18]. Other researchers mixed alginate with polymers such as pectin, chitosan, ethylcellulose, etc. [21,22,23]. These materials are a versatile platform for drug delivery due to their ability to encapsulate and protect drugs as well as ensure a sustained and temporal release and have thus generated a substantial amount of research for the delivery of either small active compounds or biopharmaceuticals [24].

Ren et al. successfully prepared several biocompatible hydrogels based on chitosan, gelatin, and dopamine as localized release systems [25]. Dopamine is an important neurotransmitter in the substantia nigra of the brain [26] and a decrease in the dopamine content can lead to Parkinson’s disease [27,28,29]. The prepared hydrogels were demonstrated to be drug delivery vehicles that can release dopamine and inflammatory drugs simultaneously in a sustained-release manner without a burst release. All the results demonstrate that these nontoxic, injectable hydrogels are excellent candidates as a long-term and sustained drug release system for the delivery of dopamine and inflammatory drugs to treat Parkinson’s disease [25].

To improve the efficiency of drug delivery and overcome the limitations caused by the presence of the polymer alone, the carrier of the system should be a hybrid composite that allows the release of the drug only in certain conditions and after receiving certain triggers such as pH, temperature, and mechanical impulse [30]. Thus, hybrid composites are being developed and researchers have shown great interest in the use of graphene as a nanoscale filler, for overcoming the above-mentioned limitations [31,32].

Graphene is one of the fillers that are being investigated; it is defined as a single layer of sp2-hybridized carbon atoms arranged in a honeycomb two-dimensional lattice array [30,33]. In the literature, it is shown to be used as a filler after its oxidation to graphene oxide (GO) and its derivatives, such as reduced-GO (rGO), because it can improve the characteristics of the carrier due to its amphiphilic properties, where the presence of oxygen functional groups allows further functionalization, and the presence of a large hydrophobic surface area allows the stabilization of hydrophobic drugs [34]. Lately, the application of pristine graphene has been investigated due to its better conductivity and mechanical stability, which can help modulate the release of the drug [35]. However, the thermal behavior of pristine graphene within a polymeric matrix has not been fully investigated yet, but the thermal triggering approach to drug delivery is common in cancer [36,37,38] and brain tumors [39,40]. Moreover, it was shown through cytotoxicity tests that graphene not only is not cytotoxic, but it can improve biocompatibility, including its ability to interact with cells, helping cell growth and improving tissue performance [41]. GO-hybridized nanogels were used for the controlled delivery of doxorubicin, an anticancer drug, showing tumor-targeting capacities, biocompatibility, and low toxicity [42]. Other researchers modified GO with carboxymethyl chitosan and subjected it to a conjugation of hyaluronic acid and fluorescein isothiocyanate to encapsulate doxorubicin. The newly formed complex targeted cancer cells and successfully inhibited their growth [43].

**Figure 1:**
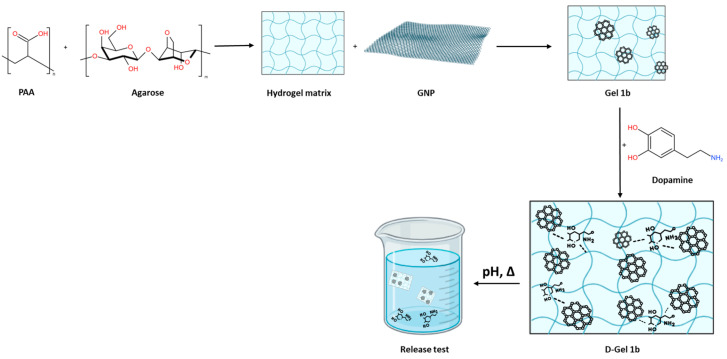
The scheme of hydrogel preparation.

The present research aimed to obtain a biocompatible hydrogel with graphene content for dopamine release (Figure 1). We chose these components of hydrogel applied for dopamine delivery based on a previous study [1] that proved no cytotoxicity of the system. Additionally, dopamine was chosen for our evaluation due to its biological and clinical significance as a neurotransmitter involved in crucial pathways related to neurodegenerative diseases, particularly Parkinson’s disease. This choice highlights the capability of the proposed hydrogel system for controlled release and its potential to deliver therapeutic agents that are otherwise rapidly degraded and poorly transported across the blood–brain barrier. The practical implications of using dopamine extend the applicability of our hydrogel to a relevant clinical context.

## 2. Materials and Methods

To contextualize the novelty of the present work, the proposed hydrogel system represents a novel advancement in drug delivery technologies, distinguished by the incorporation of exfoliated graphene within a biopolymer matrix. This integration is achieved through an innovative microwave-assisted exfoliation method, setting our material apart in the drug delivery landscape. The synthesis approach we have adopted not only streamlines the production of graphene-laden hydrogels but also imparts a unique microstructure that supports controlled drug release. This control is further fine-tuned by the well-known properties of graphene, enabling the hydrogel to respond dynamically to different changes. For example, such thermal responsiveness facilitates thermally regulated drug release, a capability not commonly observed in conventional hydrogel systems. Moreover, the contribution of graphene extends to enhancing the mechanical durability of the hydrogel, broadening its application potential across diverse biomedical scenarios. The strategic design and functional properties of the proposed hydrogel underscore its novelty and position it as a significant enhancement over current drug delivery mechanisms. It suggests new avenues for the controlled delivery of a wide array of therapeutic agents, particularly those requiring precise release modulation, thereby contributing a meaningful innovation to the realm of controlled-release formulations.

### 2.1. Materials

The gel was prepared using polyacrylic acid (PAA, Carbopol 940, Acros Organics (Geel, Belgium), MW 104,400 g/mol), agarose LE (Thermo Scientific Chemicals (Waltham, MA, USA), MW 200 kDa), dopamine hydrochloride (Merck (Darmstadt, Germany), MW 189.64 g mol^−1^), and graphene nanoplates (GNP). The GNP was produced from commercially available intercalated graphite (Asbury Carbons, Anthracite Industries, Inc., Asbury, IA, USA) by microwave irradiation. The dopamine release medium was phosphate-buffered saline (PBS, Lonza, 10× concentrated).

### 2.2. Synthesis of GNP

The GNP was obtained through the exfoliation method previously developed at the INFN NEXT Nanotechnology Laboratory in Frascati (Rome, Italy). The GPN was obtained through microwave irradiation and exfoliation methods. The graphite was put inside a ceramic container under microwave irradiation (800 W) 10 times until all the graphite particles developed a worm-like resemblance, after which those particles were exfoliated in isopropyl alcohol under pulsed-mode sonication at room temperature. The alcohol was then evaporated [44,45]. 

### 2.3. Hydrogel Preparation

The hydrogel preparation was executed following the same steps as in Mauri et al. [1]. A total of 50 mg of PAA was dissolved in 9.95 mL of distilled water, and the solution was left to stabilize for 30 min under stirring. After that, the pH of the solution was brought to a value of 7–8 by adding NaOH 1M. A total of 80 mg of agarose was added to the solution, and everything was sonicated for 10 min. After that, the solution was heated to 80 °C to induce the condensation of carboxyl and hydroxyl groups. The resulting solution was left to cool to 60 °C.

In the meantime, a solution of graphene was prepared. A total of 10 mg of graphene was sonicated in 2 mL of isopropyl alcohol until the graphene was completely dispersed, then the alcohol was evaporated at 110 °C. After the evaporation, 10 mL of distilled water was added to the graphene, and the solution was sonicated once again until the total dispersion of the graphene. Two samples were made: one with only graphene (Gel 1b) and one with graphene and dopamine (D-Gelb 1). For D-Gel 1b, 5 mg of dopamine was added to the graphene solution (drug loading). After the addition of dopamine to the solution, it was important to work in dark conditions to avoid faster dopamine degradation. As soon as the PAA and agarose solution reached 60 °C, the graphene solution was added to the initial solution. The final solution was left to cool for 6 min. The final concentrations were 0.5 mg mL^−1^ for graphene and 0.25 mg mL^−1^ for dopamine.

### 2.4. Swelling

The swelling experiments were carried out by putting a portion of lyophilized gel in 10 mL of PBS (0.1M, at pH 1.4, pH 6.8, and pH 7.2). The weight of the gel was monitored for one day, and the swelling percentage was calculated with the following equation [1]:

(1) $$\mathrm{Swelling}\;\%=\frac{W_{2}-W_{1}}{W_{1}}\times 100$$

where *W*_2_ (g) is the weight of the wet sample and *W*_1_ (g) is the weight of the dried sample.

### 2.5. Characterization

#### 2.5.1. UV-Vis Analysis

The quantification of dopamine was performed using the UV-Vis spectrophotometer TG80+ UV-Vis, PG Instruments Limited. Calibration curves for pure dopamine in PBS at pH 7.4, pH 6.8, and pH 1.4 were obtained before the experiments to calculate the concentration of dopamine released by the gel.

#### 2.5.2. Scanning Electron Microscopy (SEM) Analysis

SEM analyses were carried out using a Vega II microscope (Tescan, Brno, Czech Republic). The specimens were placed onto a microscope stub and a sputter coater (Cressington, Liverpool, UK) was used to cover specimens with a thin gold layer. Then, the specimens were introduced into the SEM chamber, and analyses were performed using a high voltage of 20 kV.

#### 2.5.3. Raman Spectroscopy 

To record the Raman spectra, an InVia microscope (Renishaw, Wotton-under-Edge, UK) was employed with a laser source set at a wavelength of 532 nm and a 100× objective. The specimens were placed onto a microscope slide. The range examined was 500–3200 cm^−1^ using an 1800 (L)/mm grating.

#### 2.5.4. Powder X-ray Diffraction (XRD)

The samples were scanned using DIFFRAC plus XRD Commander program by monochromatic CuKα1 radiation generated with a Bruker D8 Advance Diffractometer, which was equipped with an X-ray tube operating at 40 kV and 40 mA, a LINXEYE detector, and a germanium (1 1 1) monochromator. 

#### 2.5.5. Fourier Transform Infrared (FT-IR) Spectroscopy Analysis

The FT-IR spectra were recorded using a JASCO FTIR-6100 spectrometer (JASCO International Co., Ltd., Tokyo, Japan) by the KBr pellet technique in the 4000 to 400 cm^−1^ spectral range, with 4 cm^−1^ resolution. Each sample has been dispersed in about 300 mg of anhydrous KBr mixed with an agate mortar. The pellets were obtained by pressing the mixture into an evacuated die. The spectra were collected and analyzed with Jasco Spectra Manager v.2 software.

### 2.6. In Vitro Release Studies

A total of 1 mL of gel was put in 6 mL of PBS at 25 °C, 30 °C, and 37 °C. The test tube was covered with paper and aluminum foil to keep the gel in the dark. Every hour, 0.5 mL of PBS was withdrawn from the receiving medium and the same volume of fresh medium was put back in the vial to maintain sink conditions. The calculations for the cumulative release were performed using the following equation [46]:

(2) $$Q_{n}=C_{n}\times V_{0}+\sum_{i=1}^{n-1}C_{i}\times V$$

where *Q_n_* (mg) is the cumulative release of the *n*th sampling point, *C_n_* (mg/mL) is the concentration of dopamine in PBS at the *n*th sampling point, *V*_0_ (mL) is the total volume of the release medium, *V* is the volume of each sample withdrawn, and *C_i_* (mg/mL) is the concentration of the solution. The data obtained from the release tests were analyzed with different kinetic models (zero order, Higuchi, and Korsmeyer-Peppas models) to find the best-fitting one.

### 2.7. Cytotoxicity Assay

The biocompatibility of the hydrogel was assessed in vitro, using both normal cells (HaCaT) and the A375 melanoma cell line. The obtained hydrogel was dispersed in DMSO at a concentration of 0.007 g/mL. The final concentration of DMSO used on cell lines is recommended to be under 0.5%. Serial dilutions were obtained to reach the concentrations presented in the results of the experiments below.

## 3. Results

### 3.1. Hydrogel Formation and Characterization

**Figure 2:**
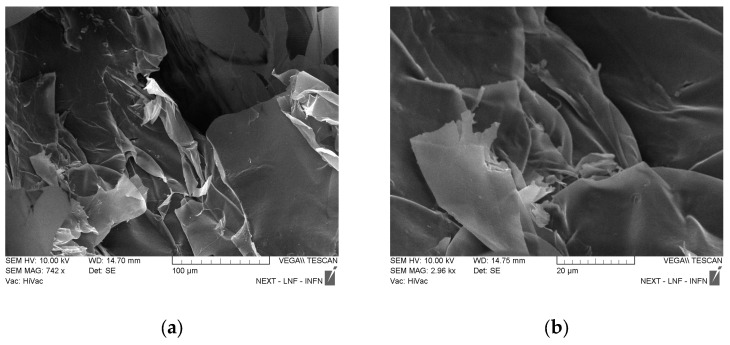

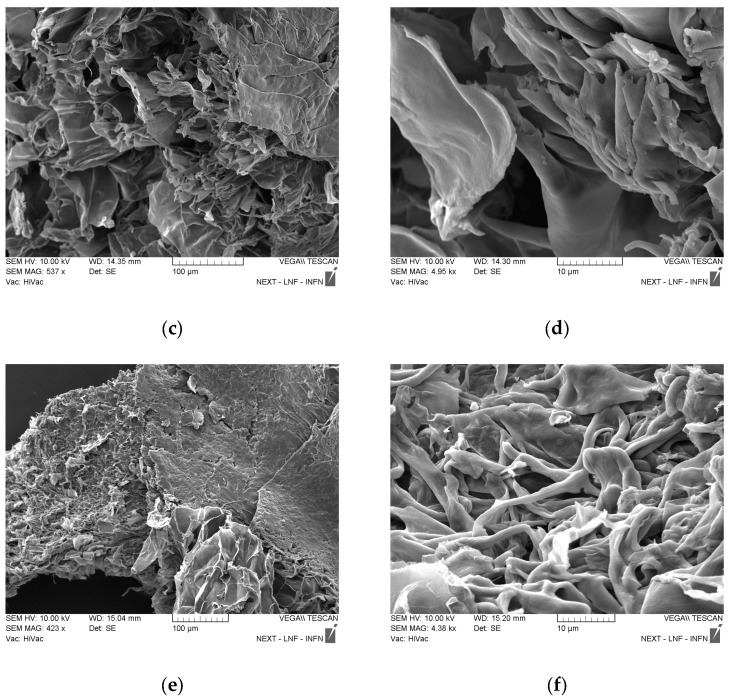
SEM images of all the samples. (**a**,**b**) PAA-agarose; (**c**,**d**) Gel 1b; (**e**,**f**) D-Gel 1b.

The hydrogel was prepared with two different polymers: PAA and agarose. The latter is a linear biopolymer extracted from red algae and made of two monomers, 1,3-linked β-D-galactopyranose and 1,4-linked 3,6-anhydro-α-L-galactopyranose. Agarose is known for its ability to form a physically crosslinked double-helix gel network when its solution in water is cooled down during the hydrogel synthesis. The 3D network obtained contains polymer chains connected through H-bond and hydrophobic interactions, and by varying the number of these interactions, the properties of the hydrogel itself can be varied [47]. The agarose chain has ionizable groups in its structure and this feature gives the polymer pH-responsive properties that are extremely useful for drug release purposes [48]. PAA (C_3_H_4_O_2_)_n_ is a synthetic biocompatible polymer, and it has been useful for multiple uses, such as drug delivery and tissue engineering. Due to its chemical structure, it was known to have excellent mucoadhesive properties and anti-inflammatory action, both good qualities for a drug delivery system [49]. When put together, the gels assemble in a mixture with excellent properties thanks to the gelation mechanism that occurs between the carboxyl groups of PAA and the hydroxyl groups of agarose. The mechanism is propelled by the temperature increase that enhances the macromer mobility and thus the inter-chain interactions [1]. The GNP was added to the gel after the heating to avoid any change in the graphene properties and a faster dopamine degradation. Dopamine was not added directly into the gel but into the graphene solution to make it bond to GNP first through π-π and physical interactions. If a good dispersion of the graphene in the polymer matrix was obtained, it also ensured a good dispersion of the drug in the sample. When the gel was cooled down, the mobility of the polymer chains drastically decreased, provoking an increase in viscosity and building the final matrix [50]. As shown in Figure 2, the polymer matrix is homogeneous and the resulting polymer sheets are thin; there is no evidence of the presence of nanostructures. The same external morphology as in Figure 2a can be observed in (c) and (e) when a similar magnification was being applied. In particular, knowing that the samples shown in (c) and (e) both have GNP, it can be observed that the graphene covered the surface and the polymers’ layers homogenously, meaning that during the gel preparation, graphene was well-dispersed in the polymer matrix. In (f), at higher magnification, a different morphology is shown compared to the similar magnification in (b) and (d), and it is attributed to the presence of dopamine in the sample, which links the GNP flakes on the different polymer planes through, as said before, π-π and physical interactions. In (f), it is evident that dopamine was successfully homogeneously dispersed in the sample.

**Figure 3:**
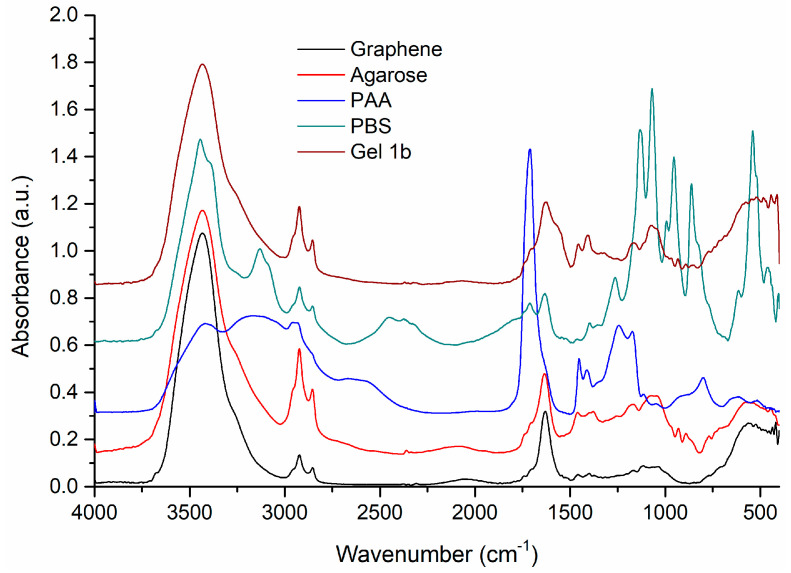
The FT-IR spectra of pure graphene, agarose, PAA, and PBS are compared with the Gel 1b sample. Spectral domain 4000–400 cm^−1^.

Further investigations on the samples were made with FT-IR. The spectra of Gel 1b and their components are presented in Figure 3.

The FT-IR spectrum of agarose shows the characteristic vibrational bands at 1170 and 1074 cm^−1^, corresponding to –C–O–C– and glycosidic linkage, and at 930 and 891 cm^−1^, assigned to absorption bands of 3,6-anhydrogalactose and C–H bending [51,52].

The spectrum of PAA presents broad bands, due to the large number of local conformations in the polymeric chains. Besides the broad bands, a strong sharp band can be observed at 1711 cm^−1^, assigned to the vibrations of the C=O group of the COOH of the monomer [53] and the intermolecular hydrogen bonds among the C=O groups of the polymer [54]. The other vibrational bands present in the spectrum can be assigned as follows: 1446 cm^−1^ (δCH_2_), 1241 cm^−1^ (δOH), 1172 cm^−1^ (νC-O), 801 cm^−1^, and 629 cm^−1^ (δC=O). In the FT-IR spectrum of graphene, the characteristic vibrational frequencies of graphene and starting graphite can be identified at 3433 and 1631 cm^−1^, corresponding to the O-H bending and C=C stretching vibrations [55]. The other bands were observed at 2923 and 2854 cm^−1^, assigned to assym. and symm. vibration of C-H bonds, and low-intensity bands at 1705sh, 1526, 1399, and 1063 cm^−1^ due to C=O, C-OH, and C-O group vibrations, respectively. The band of low intensity at 1545 cm^−1^ can be assigned to the skeletal vibration of the graphene sheets [56].

The FT-IR spectrum of the Gel 1b sample shows broad bands, characteristic of an amorphous material. In the spectrum, the vibration bands of agarose, PAA, and graphene are mostly found, these three components being the majority in the gel obtaining process. PBS was used only to adjust the pH of the solution, so it exists in small quantities, below the detection limit of the device. Thus, the main vibration bands present in the gel spectrum are 1705, 1625, 1567, 1457, 1408, 1167, 1073, 933, 892, and 851 cm^−1^. These bands, with small intensity, broadened, and slightly shifted, come mainly from the spectral contribution of agarose, PAA, and graphene.

**Figure 4:**
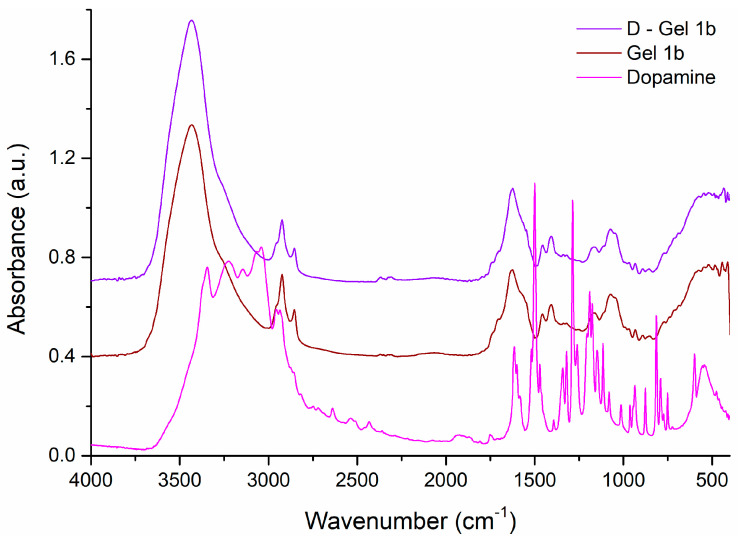
The FT-IR spectra of dopamine and Gel 1b compared to D—Gel 1b. Spectral domain 4000–400 cm^−1^.

The peaks observed in the FT-IR spectra of dopamine (Figure 4) were at 3344 cm^−1^ (amine N−H stretching), 3224 cm^−1^ (νO−H phenol), 3041 cm^−1^ (νC−H aromatic), 2935 cm^−1^ (νC−H alkyl), 1615 cm^−1^ (δN−H amine), 1519 cm^−1^ (νC=C aromatic), the region between 1200 and 1500 cm^−1^ being assigned to C–C, C–O, and C–N group vibrations, 1260 cm^−1^ (νC−N amine), and 1204 cm^−1^ (νC−O phenol) [57,58].

By adding dopamine to Gel 1b, D-Gel 1b was obtained, whose FT-IR spectrum is very similar to that of Gel 1b (caused by the small amount of dopamine used), although the presence of dopamine can be observed by the appearance of new bands of very low intensity, such as the band at 1512 cm^−1^ as a shoulder, or the shift and increase in intensity of some vibrational bands of Gel 1b in the spectrum of D-Gel 1b, shifting the bands from 1626, 1111sh, and 852 cm^−1^ to 1623, 1114, and 856 cm^−1^, respectively, and increasing the intensity of the bands at 1338 and 1319 cm^−1^.

**Figure 5:**
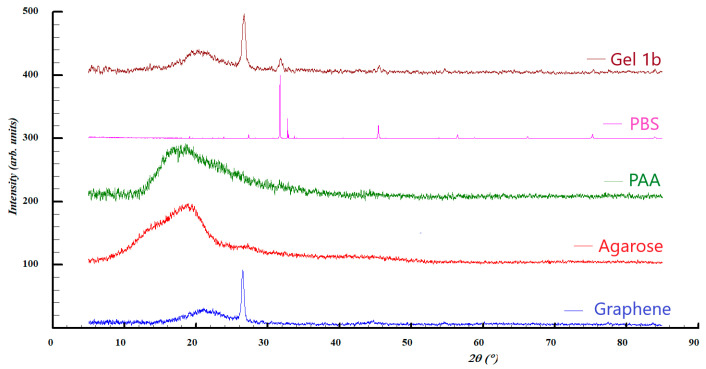
XRD patterns of Gel 1b, PBS, PAA, and graphene.

**Figure 6:**
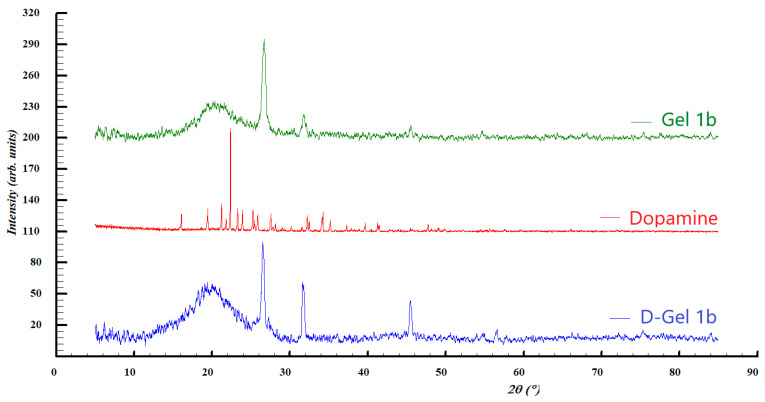
XRD patterns of Gel 1b, dopamine, and D-gel 1b.

Structural insights upon the investigated samples were also evaluated by XRD analysis, the comparison between the diffraction patterns being illustrated in Figure 5 and Figure 6.

In the case of graphene and Gel 1b, broad diffraction peaks can be observed at 2ϴ = 21°, which can be assigned to the presence of graphene with (0 0 2) Miller indices. This is consistent with the results of the Raman analysis, where the existence of a multi-layered graphene was observed. In both samples, a diffraction line is present at 2ϴ = 26.5° related to carbon materials, with a crystallinity such as graphite, characterized by (0 0 2) Miller indices. However, the pattern of Gel 1b shows additional diffraction lines at 2ϴ = 31.7° and 45.5°, which can be attributed to the crystalline phase of NaCl, which originates from the phosphate-buffered saline solution.

The patterns of PAA and agarose are present in two amorphous halos, which are centered roughly at 2ϴ = 19°.

Diffraction pattern comparison of the second set of samples suggests the presence of a mixture of graphene, by the diffraction peaks at roughly 2ϴ = 21°, and graphite, with the diffraction lines seen at 2ϴ = 26.5° in both Gel 1b and D-Gel. Once again, the diffraction peaks at 2ϴ = 31.6° and 45.4° seen for the pattern of Gel 1b and D-Gel 1b indicate that the NaCl salt is found in PBS. The diffraction pattern of dopamine showed a high degree of crystallinity which is not found in the other samples, most likely due to the small quantity. Its crystal structure can be assigned to the hydrochloride salt form of dopamine (dopamine hydrochloride) previously reported [59]. 

**Figure 7:**
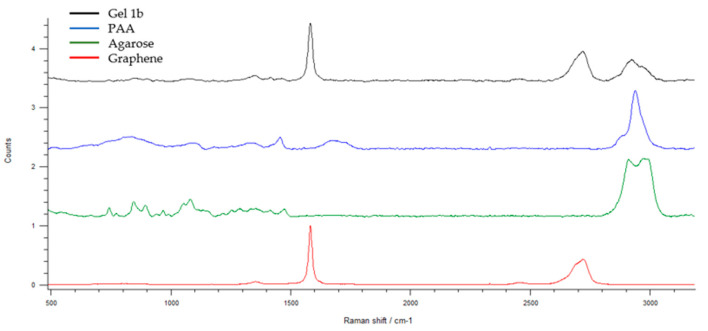
The Raman spectra of graphene, agarose, PAA, and Gel 1b. Spectral domain 500–3200 cm^−1^.

Figure 7 shows the Raman pattern of Gel 1b and its components. The presence of graphene, PAA, and agarose is confirmed by the appearance of their characteristic peaks in the Gel 1b pattern. In particular, it is possible to see the G-peak and 2D peak of GNP at 1584 cm^−1^ and 2722 cm^−1^, respectively. The appearance of weak peaks in the region between 700 and 1500 cm^−1^ and at 2900 cm^−1^ is related to the presence of PAA and agarose. 

The assignments of the Raman bands of PAA and agarose are listed in Table 1 [53,60]. 

**Figure 8:**
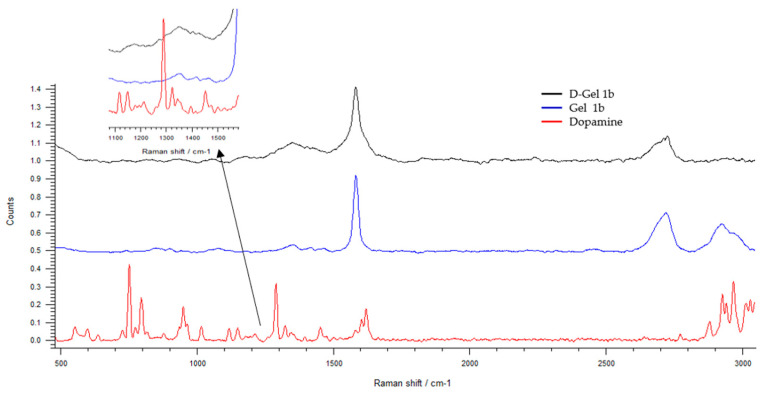
The Raman spectra of dopamine and Gel 1b compared to D—Gel 1b. Spectral domain 500–3200 cm^−1^.

In Figure 8, the Raman spectra of dopamine, Gel 1b, and D-Gel 1b are compared. The peak signal is weak due to the predominant presence of GNP; nevertheless, in the D-Gel 1b sample, it is possible to see the appearance of dopamine peaks in the range between 600 and 1000 cm^−1^. The presence of broad peaks is related to the amorphous structure of the samples investigated. 

In D-Gel 1b samples, a redshift of the G peak of GNP from 1584 to 1580 cm^−1^ is observed. This is further confirmation of the presence of GNP and their interaction with dopamine; indeed, the redshift can be attributed to charge transfer from GNP to dopamine [61].

**Figure 9:**
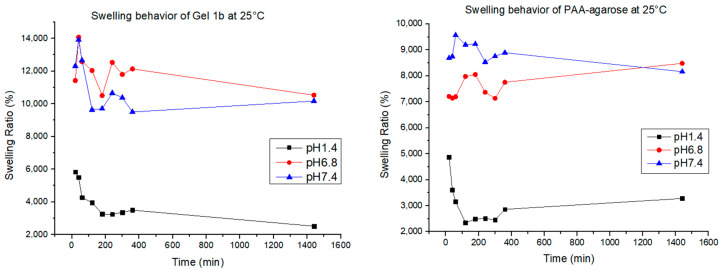

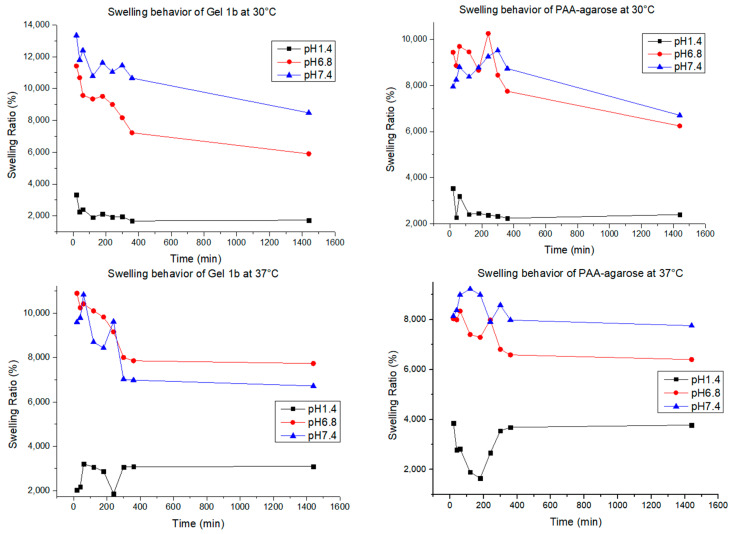
Swelling behavior for Gel 1b and PAA-agarose gel at different pH values and temperatures.

Swelling tests were carried out for Gel 1b and the gel made only with PAA and agarose. The test was carried out by putting 0.01 g of gel at 25 °C, 30 °C, and 37 °C in 10 mL of PBS at three different pH levels (1.4, 6.8, and 7.4). The swelling of the gels was monitored gravimetrically for 24 h. The results are shown in Figure 9. 

The swelling behavior of the hydrogel helps us to understand how the polymer changes its mechanical behavior when in contact with the medium. Hydrogels are known for their capacity to retain a high amount of water, ensuring the exchange and diffusion of molecules with the media [1]. The water uptake of every hydrogel is conditioned by many factors, such as the charge of electronic groups, the pore size, and the hydrophilicity [62], and in our case, for the hydrogel that contains GNP, it can also be influenced by the interaction between the matrix and the graphene. PAA, as said before, is known as a highly swellable polymer due to the presence of a high number of charges over the polymeric chains, making it a polymer able to swell at all pH values; agarose, on the other hand, has good swelling abilities mostly at neutral and basic pH due to the appearance of negative charges [63]. In all the samples, the quantity of agarose is more than the quantity of the other elements, and that explains why the swelling percentage for all the samples at acidic pH is always lower, at any temperature. For the samples of PAA-agarose gel, it can be observed that for pH 6.8 and 7.4, there is not a big difference in swelling at different temperatures, while for the samples with graphene, there is a bigger difference in swelling; therefore, the presence of GNP results in a temperature-dependent modulation of the hydrogel, as also found in the work of Mauri et al. [1]. In our case, it was also found that the hydrogel could swell and de-swell, a good characteristic for a drug delivery system, since it can help maintain a more constant release of the drug over time [64]. 

### 3.2. Drug Release Tests

The first release test of D-Gel 1b was carried out in different temperatures—25 °C, 30 °C, and 37 °C—and in the same media (PBS 0.1 M, pH 7.4). The volume in every vial was 1 mL of hydrogel in 6 mL of media. We took aliquots of media (500 μL) at 1 h, 2 h, 3 h, 4 h, 5 h, 6 h, 22 h, 23 h, and 24 h. After taking the aliquot of media, fresh PBS of the same volume was put back to keep sink conditions. The absorbance of dopamine released (characteristic peak λ ≈ 280 nm) in solution was registered using a UV-Vis spectrophotometer, and then the concentration values were interpolated in a calibration curve to obtain the concentration of dopamine released at every sampling. 

Dopamine (3-hydroxytyramine) is a neurotransmitter of the family of the catechols that plays a vital role in both mental and physical health in humans [65]. Its absence or excess can cause many diseases of different kinds, and this is why, in recent years, drug delivery systems, mostly made of hydrogel because of their similarities with the extracellular matrix, have been studied to find therapies [25]. Free dopamine is a very unstable molecule due to its thermal and photo sensibility, and during the release process, auto-oxidation and polymerization occur, causing dopamine to transform into polydopamine, melanin, and its derivatives [66]. 

Different temperatures were studied for the release to investigate their potential effects on the drug release system. As shown in Mauri et al. [1], the release profile of the drug in the PAA-agarose hydrogel is not related to a change in temperature, while it was when graphene was introduced into the gel matrix due to the presence of physical interactions between the drug (diclofenac) and the graphene itself. In our work, we investigated if the release of dopamine from the hydrogel could be influenced by the pH of the receiver, so other release tests were carried out at 37 °C for D-Gel 1b at pH 1.4, 6.8, and 7.4.

**Figure 10:**
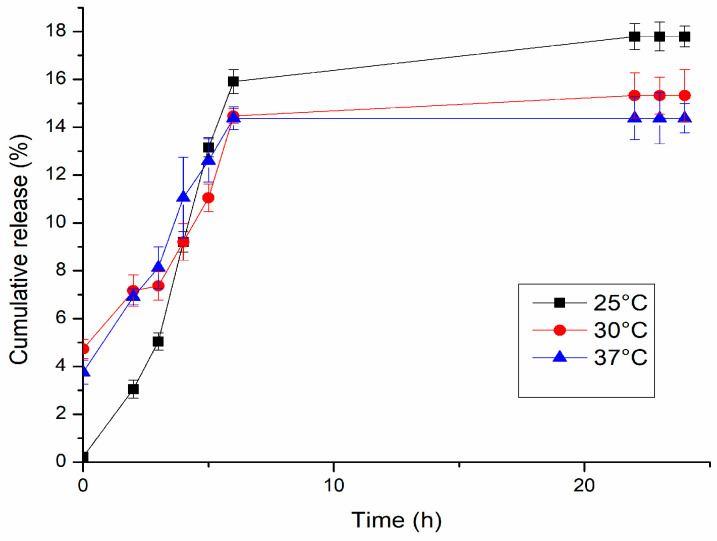
Dopamine release profiles of D-Gel 1 in PBS pH 7.4 at different temperatures.

**Figure 11:**
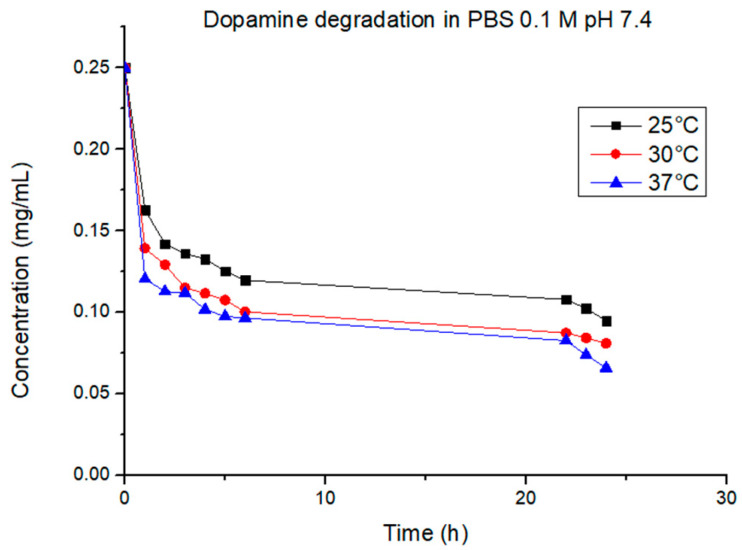
Dopamine degradation at different temperatures.

The cumulative drug release percentage obtained in the first part of the experiments at 25 °C, 30 °C, and 37 °C is shown in Figure 10. In our case, due to the dopamine sensibility, is not possible to establish with precision the correlation between the temperature and the quantity of drug released. At 25 °C, the total quantity released (17.8%) was more than at 30 °C (15.3%) and 37 °C (14.4%). This should prove that the release from the hydrogel–graphene matrix was not thermally triggered, but with the increasing temperature, there was an increasing degradation rate of dopamine. The degradation rate of dopamine at the chosen temperatures was studied to better understand the release mechanism of the gel matrix prepared. As shown in Figure 11, the concentration of dopamine in solution was strongly correlated to the temperature. For the sample at 25 °C, it was noticeable that the dopamine degradation proceeded at a much slower rate compared to 30 °C and 37 °C; after 6 h at 25 °C, the dopamine left in solution was 47% of the initial amount, while at 30 °C and 37 °C, it was 40% and 38%, respectively. After 24 h, the final concentration of dopamine registered was 38% at 25 °C, 32% at 30 °C, and 26% at 37 °C, with respect to the initial concentration. At all temperatures measured, the best-fitting model of the degradation process was found to be the first order, with R^2^ > 0.92. The rate constant was 0.024 mg/h∙mL at 25 °C, 0.027 mg/h∙mL at 30 °C, and 0.020 mg/h∙mL at 37 °C. The half-life was 12.5 h, 10.9 h, and 14.8 h, respectively.

The data obtained from the release were studied with three models of kinetic release (zero order, Higuchi, and Korsmeyer-Peppas models) to find the best-fitting one; this was needed to identify the release mechanisms of the system.

In Table 2, the R^2^ parameter for D-Gel 1 is shown for every kinetic model studied; R^2^ is the coefficient of determination, which was used to evaluate the best model to fit the data. In the first 6 h and for all the temperatures, the best model found was the zero-order model, but after 6 h, the release reached a plateau due to different complex factors, such as the structural characteristics of the hydrogel, the release environment, the dopamine sensibility to heat and pH, and all the possible interactions between all these factors [67]. Thus, the pseudo-zero order model was the more suitable model. For these models, the release of the drug happens at a nearly constant rate, and it can be described by the following equation [68]:

(3) $$Q_{t}=Q_{0}+k_{0}\times t$$

where *Q_t_* (mg) is the amount of drug dissolved at time *t*, *Q*_0_ (mg) is the initial amount of drug in the solution, and *k*_0_ (h^−1^) is the zero-order release constant. In this model, the release of the drug was only a function of time, and the process took place at a constant rate, independent of drug concentration due to the release that was governed by factors like diffusion of the drug through a matrix [69]. The pseudo and zero-order models can be considered advantageous models for the release of drugs thanks to their characteristics, which ensure a constant and consistent release rate, resulting in less probability of side effects and reduction in drug administration, unlike conventional drug administration, which often requires high dosages or repeated administration to stimulate a therapeutic effect [70].

At 30 °C and 37 °C, the R^2^ registered for the Higuchi fitting was comparable to the pseudo-zero order. This model is based on Fick’s law of diffusion and is represented by the following equation [68]:

(4) $$Q_{t}=k_{H}\times t^{1/2}$$

where *Q_t_* (mg) is the amount of drug dissolved at time *t* and *k_H_* (mg∙h^−1/2^) is the Higuchi constant. In this model, the constant of proportionality has a specific realistic meaning and is determined by the properties of the matrix (thickness and surface area) and the diffusivity of the drug [69]. The application of this model was useful in designing drug delivery systems to provide a more convenient release of the drug by simply modifying the systems’ properties [71]. 

**Figure 12:**
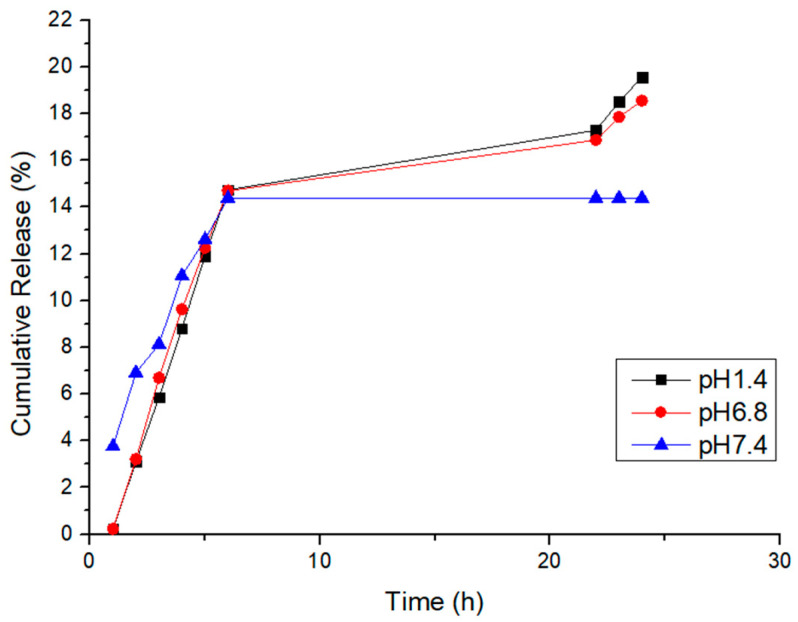
Cumulative release of D-Gel 1b at 37 °C at different pH values.

In Figure 12, we delve deeper into the dopamine release profiles from D-Gel 1b at 37 °C across different pH levels. The initial release rates within the first 6 h appear to be pH-independent. However, a prolonged release is observable under acidic conditions (pH 1.4) beyond 24 h, suggesting that the acidic environment may enhance the stability of dopamine or affect the interaction with the hydrogel matrix. This finding indicates that the release mechanism is not solely governed by diffusion but may also involve pH-sensitive binding within the hydrogel. This nuanced understanding points to the potential of the proposed hydrogel for tailored release in various organ environments, each characterized by distinct pH levels. We propose that the extended release in acidic conditions could be due to additional physical or chemical interactions that warrant further investigation. The observed trend underscores the importance of environmental pH in influencing drug release kinetics and supports the development of our hydrogel system for applications requiring sustained release, particularly in acidic bodily compartments. These outcomes evidence the necessity for continuing this line of inquiry in subsequent studies.

The kinetics of this release test were analyzed following the same passages carried out in the first release test; the results are shown in Table 3. Once again, the release kinetics of the gel followed the pseudo-zero order, meaning that with this type of hydrogel, it was possible to obtain a good release regardless of the temperature and the pH of the release media. 

For this release, good R^2^ values were found at pH 1.4 and 6.8, and also for the Korsmeyer-Peppas model. This model is based on the following equation [68]:

(5) $$\frac{M_{t}}{M_{\alpha }}=K\times t^{n}$$

where *M_t_*/*M_α_* is a fraction of the drug released at time *t*, *K* is the rate constant incorporating structural and geometric characteristics of the delivery system, and *n* is the release exponent, indicative of the mechanism of transport of drug through the polymer. The *n* value was used to characterize different release mechanisms. In our case, *n* < 0.5, meaning that the release mechanism was a quasi-Fickian non-swellable diffusion [68].

### 3.3. Cytotoxicity

**Figure 13:**
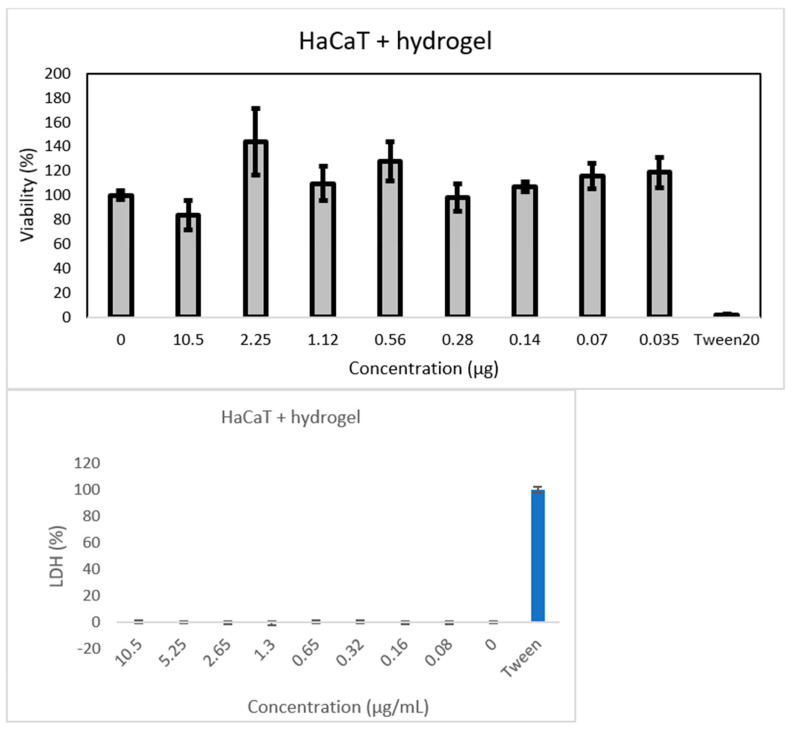
Results of MTT assay performed on HaCaT cells supplemented with hydrogel.

The potential toxicity of the synthesized hydrogel was assayed on hydrogel extracts in cell culture medium, using two different cell lines. The results in Figure 13 confirm the good biocompatibility for normal cells. The IC50 values determined are as follows:

55.04 µg/mL for HaCaT;
12.2 µg/mL for A375.

**Figure 14:**
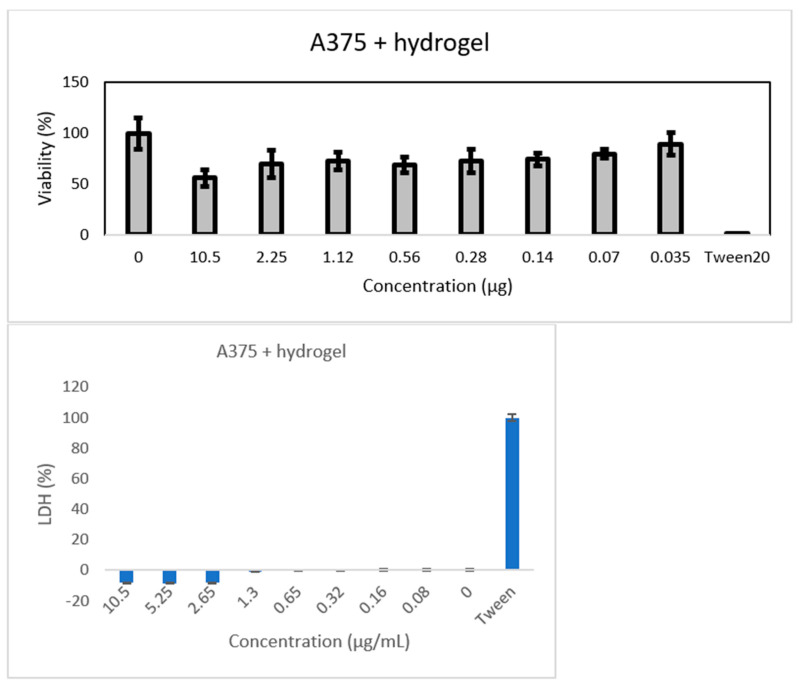
Results of MTT assay performed on A375 cells supplemented with hydrogel.

This indicates that the hydrogel is more toxic for the A375 cells (see Figure 14) compared to the HaCaT cells, which will allow the use of higher doses for treatment (between 12 and 55 µg/mL).

The overall viability of normal cells (HaCaT) was not affected, as compared to the melanoma cells, where the registered viability was under 100% control.

No LDH release was detected.

Hydrogel biomaterial extracts at different titers are shown. Cell viability was normalized to that of non-treated control cells.

Hydrogel biomaterial extracts at different titers are shown. Cell viability was normalized to that of non-treated control cells.

## 4. Conclusions

In this work, the preparation of a drug delivery system based on a hydrogel and graphene matrix was successfully executed through confirmation by different characterization methods. The GNP was found to be homogeneously dispersed over the polymer surface, ensuring a good dispersion of the dopamine that was loaded over the polymer surface. The dopamine release from the system was confirmed through release tests that were executed at different temperatures and pH values. Dopamine presence was observed even after 24 h of release, meaning that the system can slow down the drug’s degradation due to the interactions with the GNP and the polymer matrix. The dopamine release kinetics resulted in being of pseudo-zero order, which is a model optimal for drug delivery. 

Thus, the obtained hydrogel has the potential to be used in healthcare scenarios where drug release systems are needed. Specifically, the proposed hydrogel system has promising applications in precision-targeted drug delivery, with particular suitability for localized, temperature-regulated medication release in postoperative settings. Its robust composition makes it an excellent candidate for transdermal patches, facilitating controlled and sustained drug delivery critical for chronic disease management. Additionally, the integration of graphene not only bolsters the mechanics of the hydrogel integrity but also opens up prospects for electro-responsive drug delivery systems, representing an innovative frontier in precision therapeutics. In conclusion, we advocate the innovative use of few-layered graphene in hydrogels as a platform for thermally tunable drug release. The interactions between graphene, the polymeric matrix, and the drug play a crucial role in achieving controlled drug release, and such systems have the potential for various healthcare applications.

## Figures and Tables

**Table 1:** Raman assignment values for PAA and agarose.

Wavenumber (cm^−1^)	PAA
	Assignment
1674	ν (C=O) dimer
1453	δ (CH_2_)
1325	ν_as_ (CCO)
1100	ρ (CH_2_)
841	ν_s_ (CCO), τ (CH_2_)
500	δ (CCO) cis
	**Agarose**
**Wavenumber (cm^−1^)**	**Assignment**
730–960	CH deformation
1000–1160	C-O stretching
1200–1460	CH, OH deformation stretching
2900	CH stretching
3050	OH stretching

**Table 2:** Regression coefficients and kinetic parameters for zero-order, Higuchi, and Korsmeyer-Peppas models to pH 7.4 at different temperatures in the first 6 h.

Regression Coefficients				
Sample		Zero Order, R^2^	Higuchi, R^2^	Korsmeyer-Peppas, R^2^
	25 °C	0.9639	0.7839	0.9075
D-Gel 1b	30 °C	0.9476	0.9465	0.7003
	37 °C	0.9769	0.9691	0.7938
**Kinetic Parameters**				
**Sample**		**Zero Order, *k*_0_** **(h^−1^)**	**Higuchi, *k*_*H*_** **(mg∙h^−1/2^)**	**Korsmeyer-Peppas, *n***
	25 °C	2.85	0.119	0.048
D-Gel 1b	30 °C	2.07	0.177	0.064
	37 °C	2.32	0.163	0.061

**Table 3:** Regression coefficients and kinetic parameters for zero-order, Higuchi, and Korsmeyer-Peppas models at 37 °C at different pH in the first 6 h.

Regression Coefficients				
Sample	pH	Zero Order, R^2^	Higuchi, R^2^	Korsmeyer-Peppas, R^2^
	1.4	0.9791	0.8157	0.9429
D-Gel 1b	6.8	0.9814	0.8337	0.9636
	7.4	0.9769	0.9691	0.7938
**Kinetic Parameters**				
**Sample**	**pH**	**Zero Order, *k*_0_** **(h^−1^)**	**Higuchi, *k*_*H*_** **(mg∙h^−1/2^)**	**Korsmeyer-Peppas, *n***
	1.4	2.62	0.132	0.054
D-Gel 1b	6.8	2.66	0.132	0.054
	7.4	2.32	0.163	0.061

## Data Availability

Data are contained within the article.
